# Multi Texture Analysis of Colorectal Cancer Continuum Using Multispectral Imagery

**DOI:** 10.1371/journal.pone.0149893

**Published:** 2016-02-22

**Authors:** Ahmad Chaddad, Christian Desrosiers, Ahmed Bouridane, Matthew Toews, Lama Hassan, Camel Tanougast

**Affiliations:** 1 Laboratory for Imagery, Vision and Artificial Intelligence, École de Technologie Supérieure, Montréal, Québec, Canada; 2 Laboratory of Conception, Optimization and Modelling of Systems, University of Lorraine, Metz, Lorraine, France; 3 School of Computing, Engineering and Information Sciences, Northumbria University, Newcastle, United Kingdom; National Cancer Center, JAPAN

## Abstract

**Purpose:**

This paper proposes to characterize the continuum of colorectal cancer (CRC) using multiple texture features extracted from multispectral optical microscopy images. Three types of pathological tissues (PT) are considered: benign hyperplasia, intraepithelial neoplasia and carcinoma.

**Materials and Methods:**

In the proposed approach, the region of interest containing PT is first extracted from multispectral images using active contour segmentation. This region is then encoded using texture features based on the Laplacian-of-Gaussian (LoG) filter, discrete wavelets (DW) and gray level co-occurrence matrices (GLCM). To assess the significance of textural differences between PT types, a statistical analysis based on the Kruskal-Wallis test is performed. The usefulness of texture features is then evaluated quantitatively in terms of their ability to predict PT types using various classifier models.

**Results:**

Preliminary results show significant texture differences between PT types, for all texture features (*p*-value < 0.01). Individually, GLCM texture features outperform LoG and DW features in terms of PT type prediction. However, a higher performance can be achieved by combining all texture features, resulting in a mean classification accuracy of 98.92%, sensitivity of 98.12%, and specificity of 99.67%.

**Conclusions:**

These results demonstrate the efficiency and effectiveness of combining multiple texture features for characterizing the continuum of CRC and discriminating between pathological tissues in multispectral images.

## Introduction

Colorectal cancer (CRC) is a common malignancy having an increasing incidence in many developed countries. It is the third most common newly diagnosed cancer, accounting for 8% of new cases each year, and also the third most common cause of cancer death in both men and women [1]. An estimated 26,270 men and 24,040 women died of colorectal carcinoma in 2014, as reported by the American Cancer Society. Surgical resection of the primary tumor with curative intent is possible in only 70% of patients [2, 3]. Unfortunately, up to 30% of CRC patients who undergo surgical resection of the primary tumor experience a subsequent relapse within three years, with a median time to death of 12 months [4]. Imaging studies are frequently used to evaluate patients for the screening and staging of colorectal cancer. Cross sectional imaging techniques like computed tomography (CT) [4], magnetic resonance imaging (MRI) [5] and microscopy provide anatomical and morphological information about the structure and patterns of the tumor [6]. In this information, image texture, and in particular texture heterogeneity, is a prominent feature of CRC that manifests itself as areas of high cell density.

A recent computational trend has been the analysis of high-resolution whole slide images produced from digital pathology slides [7, 8]. Texture features extracted from such images serve as an input to important computational applications such as computer-aided diagnosis from pathology. An early study by Esgiar et al. showed that entropy texture features extracted from gray-level co-occurrence matrices (GLCM) were capable of differentiating between normal and cancerous tissue [6]. A follow up study by the same authors incorporated fractal dimensions into the feature analysis to improve the sensitivity and specificity of classification [9]. Using color channel histograms, GLCM and structural features, Kalkan et al. achieved an accuracy of 75.15% in the classification of four types of colon tissues: normal, cancerous, adenomatous and inflammatory [10]. Jiao et al. proposed a method for automatic colon cancer detection, using GLCM for texture extraction and support vector machines (SVM) for classification. This method achieved an accuracy of 96.67% in differentiating between cancerous and non-cancerous images [11]. Hilado et al. used 2D discrete wavelet (DW) transform features to classify whole slide colon cancer images into normal, cancerous and adenomatous polyp cases, reporting a 91.11% accuracy [12]. Francesca et al. used whole tumor texture features, computed using Laplacian-of-Gaussian (LoG) filters to assess the heterogeneity of CRC [4]. In a later study, Rao et al. considered LoG texture features to discriminate between CRC patients with and without hepatic metastases [13]. Various approaches using local descriptors have also been proposed, including methods based on scale-invariant feature transform (SIFT) [14], shape context [15], and histograms of oriented gradient (HOG) descriptors [16]. Because they rely on key points that may vary from one subject to another, key point-based SIFT and shape context features methods are not always suitable for the assessment of cellular abnormalities from optical microscopy systems. Likewise, methods based on HOG are not invariant to rotations that occur in microscopic images. There are thus strong arguments supporting the use of rotation-invariant texture features derived from GLCM, LoG filters and DW for this specific problem.

**Fig 1:**
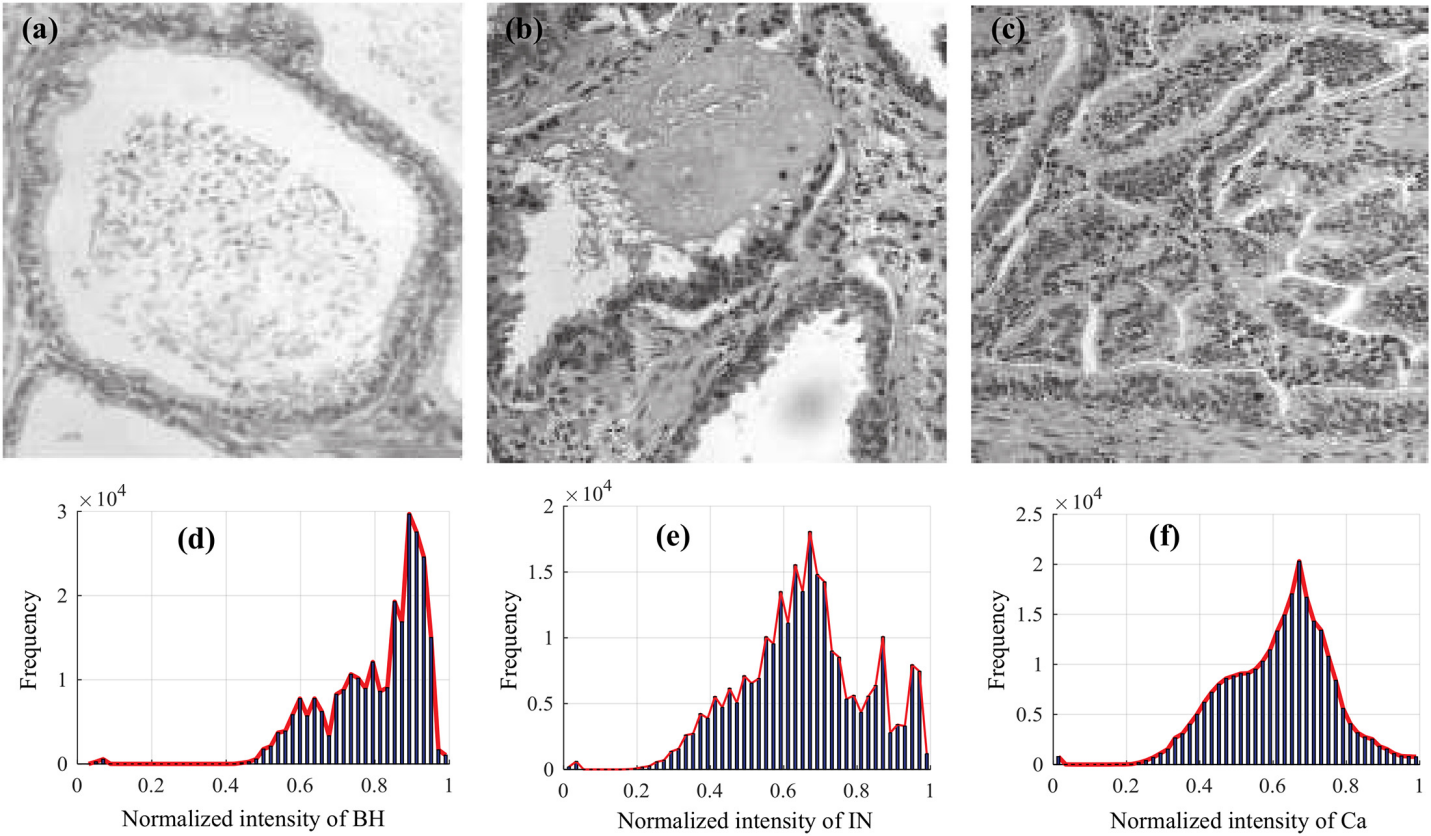
Images of three different types of pathological tissues. (a) Benign Hyperplasia; (b) Intraepithelial Neoplasia; (c) Carcinoma; (d, e, and f) Histograms show pixel intensity distributions for each type.

As a general methodology, pathological tissues (PT) resulting from cellular abnormalities in CRC, such as benign hyperplasia (BH), intraepithelial neoplasia (IN) and carcinoma (Ca), can be detected from classical optical microscopy systems using a variety of image processing techniques [17–19]. This study proposes to model the continuum of CRC using rich, informative texture features obtained from multispectral optical microscopy images. The discriminative ability of texture features can be appreciated from Fig 1, where the histogram of pixel intensities is shown for images of the BH, IN and Ca types. It can be seen that BH, IN and Ca tissues exhibit noticeably different intensity profiles, supporting the idea that such feature can be used to differentiate between these PT types. The novelty of this work lies in the comparative analysis and combination of three different texture features based on GLCM [17], LoG [4] and DW [20], for predicting PT types. As will be shown in our experiments, using multi-textural information can improve the detection and classification of pathological tissues, and provide a more comprehensive understanding of the connection between CRC and tissue heterogeneity. The potential impacts of this work on the improvement of medical care are two-fold. In combination with standard screening approaches for CRC, the proposed method could improve the detection of the disease in its early stages, thereby increasing the chances of successful treatment. According to the American Cancer Society, the 5-year relative survival rate is about 90% when CRC can be detected before it has spread [21]. The classification of cellular abnormalities in pathological tissues is also essential to assess the progression of CRC and select the appropriate course of treatment. By using texture features, our method provides an effective way of characterizing tissue properties at the cellular level. Measuring the textural information at various points in time could help track the disease’s progression and evaluate the efficiency of a given treatment.

## Materials and Methods

This study was approved by the institutional review board of the Anatomical pathology (Anapath) Department at the CHU Nancy-Brabois Hospital. The review board waived the need for written informed consent from the participants. Part of the data was used in previous studies [18, 22, 23].

**Fig 2:**
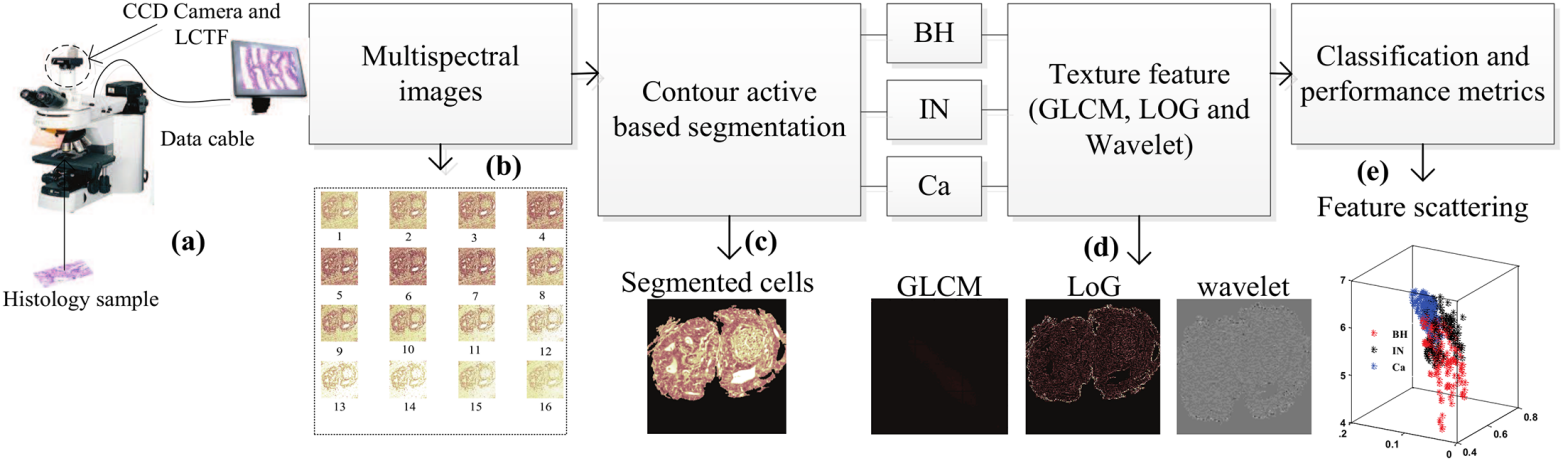
Image analysis processing pipeline: multispectral image acquisition, contour-based segmentation of abnormal tissues, texture feature extraction and finally classification. (a) Optical microscopy system, staining, sectioning, and scanning. (b) Multispectral image acquisition via a CCD camera across a range of visual spectral bands. (c) Active contour segmentation algorithm for delineating ROIs. (d) GLCM, LoG and DW image texture feature extraction. (e) Supervised classification for automatic prediction of abnormal tissue types from new samples.

The proposed framework, shown in Fig 2, consists in a series of five steps: 1) sample preparation and image acquisition, 2) ROI segmentation, 3) texture feature extraction, 4) PT type classification, and 5) performance evaluation. A detailed presentation of each step is given in the following sections.

### Sample preparation and image acquisition

CRC data were collected from the Anatomical pathology (Anapath) Department at the CHU Nancy-Brabois Hospital. Tissue samples were obtained from sequential resections of colons from 30 patients with colorectal cancer. Sections of 5 μm thickness were extracted and stained with the widely used haematoxylin and eosin (H&E) stains, to reduce image processing requirements. Images were captured at low magnification (× 40) using an optical microscope mounted with a charge coupled device (CCD) camera. A Liquid Crystal Tunable Filter (LCTF) was placed in the optical path between the light source and the CCD camera, providing a bandwidth accuracy of 5 nm and a controllable wavelength through the visible spectrum from 500 to 650 nm [24]. This technique, known as hyperspectral or multispectral imaging [25], can capture images of tissue samples at different spectral frequencies. In this study, 16 multispectral bands were acquired in the wavelength range of 500–650 nm, with 9.375 nm steps between successive bands.

The data of 30 CRC patients (10 BH patients, 9 IN patients and 11 Ca patients) were obtained for analysis, giving a total of 160 BH images, 144 IN images, and 176 Ca images. Data are available in S1 Zip (https://figshare.com/s/5e9c65848bb0aa1f4032 or DOI: 10.6084/m9.figshare.2076220). The identification of each PT type from multispectral images was done by a senior histopathologist, confirming the diagnosis. Finally, images were denoised using an average filter and rescaled to a resolution of 512 × 512 pixels.

### Active contour based segmentation

Active contour segmentation was used to identify tissue boundaries within the image. This segmentation technique, which moves a dynamic curve iteratively towards object contours in the image, is well adapted to delineate irregular shapes [26, 27]. While it can achieve a high accuracy, it can also suffer from long runtimes. To accelerate the segmentation process, we limited the number of iterations based on empirical calculations [17]. A multi-scale approach was used to further reduce runtimes by performing an initial segmentation at a coarse resolution of 64 × 64 pixels and then refining this solution at a high resolution of 512 × 512 pixels. Using this technique, segmentations were obtained in less than a minute with a standard PC (Intel Core i5 3.4 GHz processor with 32 GB RAM). Note that the runtime performance can be improved via alternative specialized computational technologies, such as a pipeline algorithm based on field programmable gate array (FPGA) technology [28].

**Fig 3:**
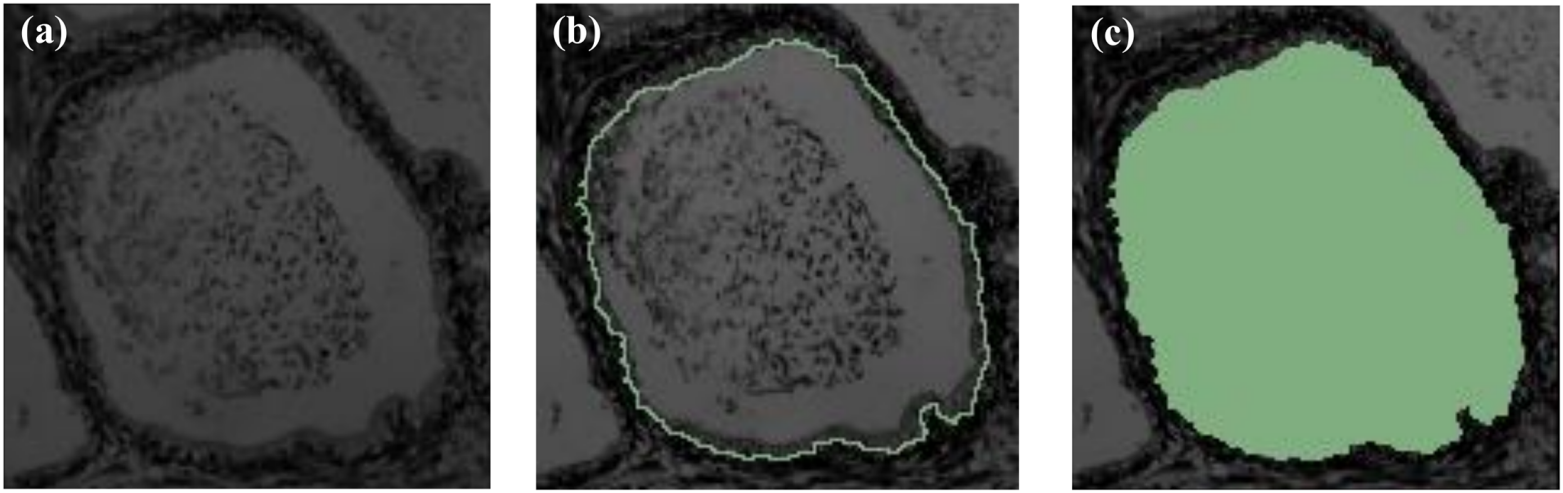
Examples of ground truth segmentation. (a) Original image. (b) Segmented image. (c) Labeled image. Labeled area in (c) corresponds to the ROI used for texture feature extraction.

**Fig 4:**
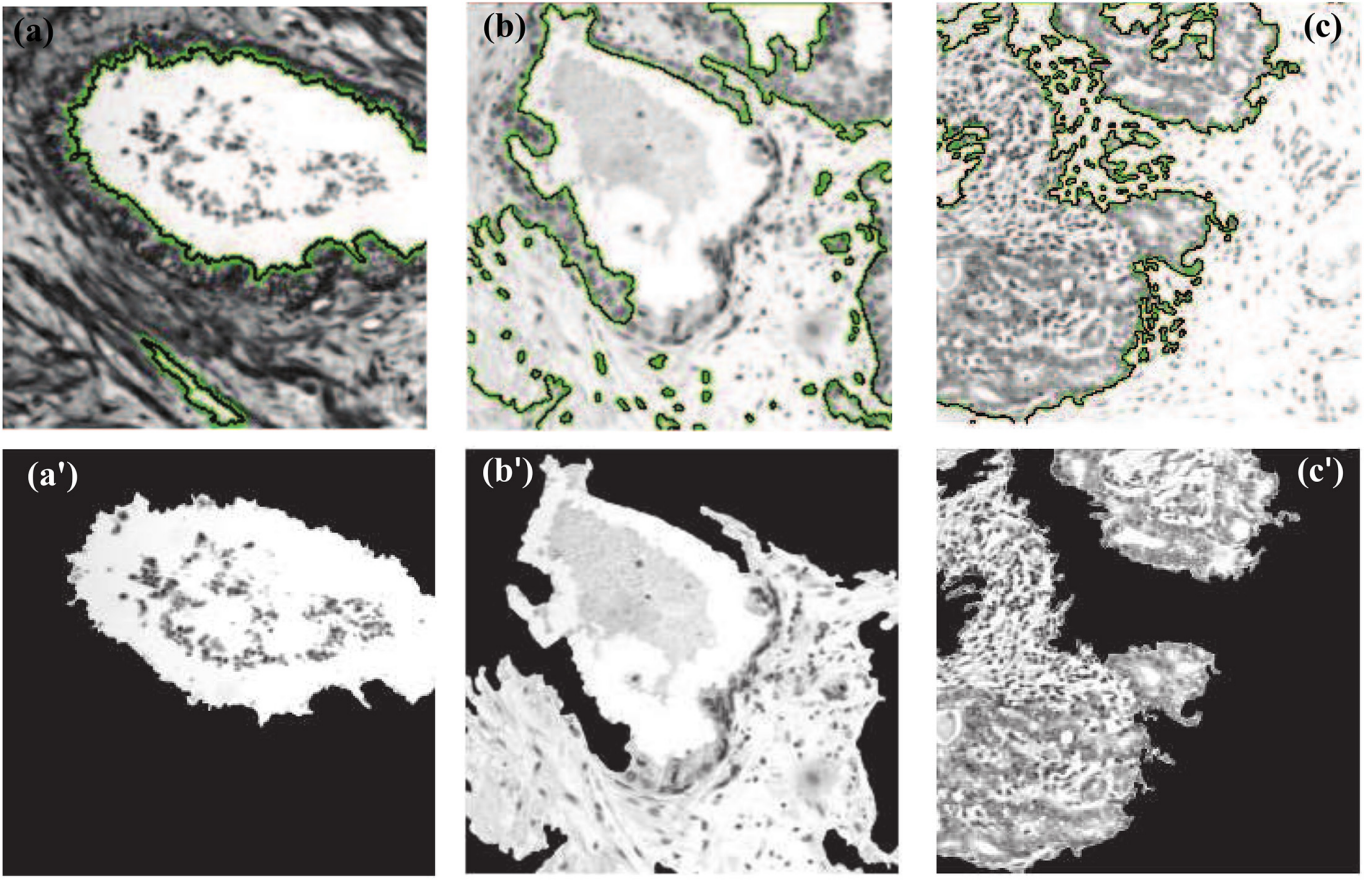
Examples of active contour segmentation. Top-row images correspond to the (a) BH, (b) IN and (c) Ca types. Bottom-row images (aˊ), (bˊ), and (cˊ) show the ROI obtained on these images by the active contour segmentation method.

Ground truth segmentations, one segmentation per sample, were obtained manually using 3D Slicer [29] and validated by two pathologists. An example of a ground truth segmentation is given in Fig 3. The ground truth images were used to evaluate the performance of the active contour segmentation (Fig 4), based on the following metrics: Dice similarity coefficient (DSC), false positive rate (FPR) and false negative rate (FNR). DSC measures the degree of the correspondence (similarity) between ground truth and segmented ROIs, and is defined as

DSC(A,B)=2|A∩B|/(|A|+|B|) (1)

where *A* and *B* are pixel sets corresponding to ground truth and segmented regions, respectively.

The FPR and FNR are two measures of over and under segmentation, and are calculated as follows:

$$\text{FPR}\left(A,B\right)=\left|B\backslash A\right|/\left|\bar{A}\right|$$ (2)

FNR(A,B)=|A\B|/|A| (3)

where $\bar{A}$ contains the pixels that are not in the ground truth set *A*.

### Texture feature extraction

Three types of texture filters, based on LoG, DW and GLCM, were applied to the segmented ROIs in each spectral band. Texture features were then extracted from the filtered images by applying specialized quantifier functions (Fig 2).

#### LoG based Texture

The Laplacian of Gaussian (LoG) filter can be viewed as the combination of a Gaussian smoothing operator with a kernel of width sigma (*σ*) and an isotropic filter, the Laplacian, which measures the second spatial derivative in the image. The LoG is commonly used to detect edges and blobs at various scales. For each segmented ROI, a LoG filter was applied using *σ* values of 0.5 (fine texture type), 1.5 (medium texture type) and 2.5 (coarse texture type). The region was then quantified by computing the Average (*A*), Standard deviation (*SD*) and Entropy (*Ent*) of its LoG values. The theoretical framework of this technique is described in detail in the Appendix B of Ganeshan et al [30]. Let *f*(*x*,*y*) be the LoG value of a pixel (*x*,*y*) in the segmented region *Ω*. For entropy calculation, we discretize the distribution of LoG values into 256 equal-sized intervals and denote as *Ω*_*k*_ the subset of pixels within the *k*-th interval. The LoG quantifier functions can be defined as follows:

$$A=\frac{1}{\left|\Omega \right|}\sum f\left(x,\;y\right)$$ (4)

$$SD=\sqrt{\frac{\sum {\left(f\left(x,\;y\right)\;-\;A\right)}^{2}}{\left|\Omega \right|-1}}$$ (5)

$$Ent=-\Sigma_{k}\frac{\left|\Omega_{k}\right|}{\left|\Omega \right|}\text{log}\frac{\left|\Omega_{k}\right|}{\left|\Omega \right|}$$ (6)

For each sample, we thus obtained a set of 9 texture:

$$F_{LoG}=\left\{A_{f},SD_{f},Ent_{f},A_{m},SD_{m},Ent_{m},A_{c},SD_{c},Ent_{c}\right\}$$ (7)

where *f*, *m*, and *c* represent the fine, medium and coarse texture.

#### Texture based on Discrete Wavelet (DW) Transform

The DW Transform (DWT) analyzes an image by decomposing it into a coarse approximation via low-pass filtering and a detailed component via high-pass filtering. The decomposition is performed recursively on the low-pass approximation coefficients obtained at each level, until the necessary number of iterations is reached [31]. Four decomposition directions (sub-bands) are considered: horizontal (0°, D_h_), first diagonal (45°, D_d_), vertical (90°, D_v_) and second diagonal (135°, D_d_). The decomposition at the each level *i* provides an approximation matrix (image) A_i_ and three detail matrices, namely, Dh_i_ (horizontal coefficient matrix), Dv_i_ (vertical coefficient matrix) and Dd_i_ (diagonal coefficient matrix).

**Fig 5:**
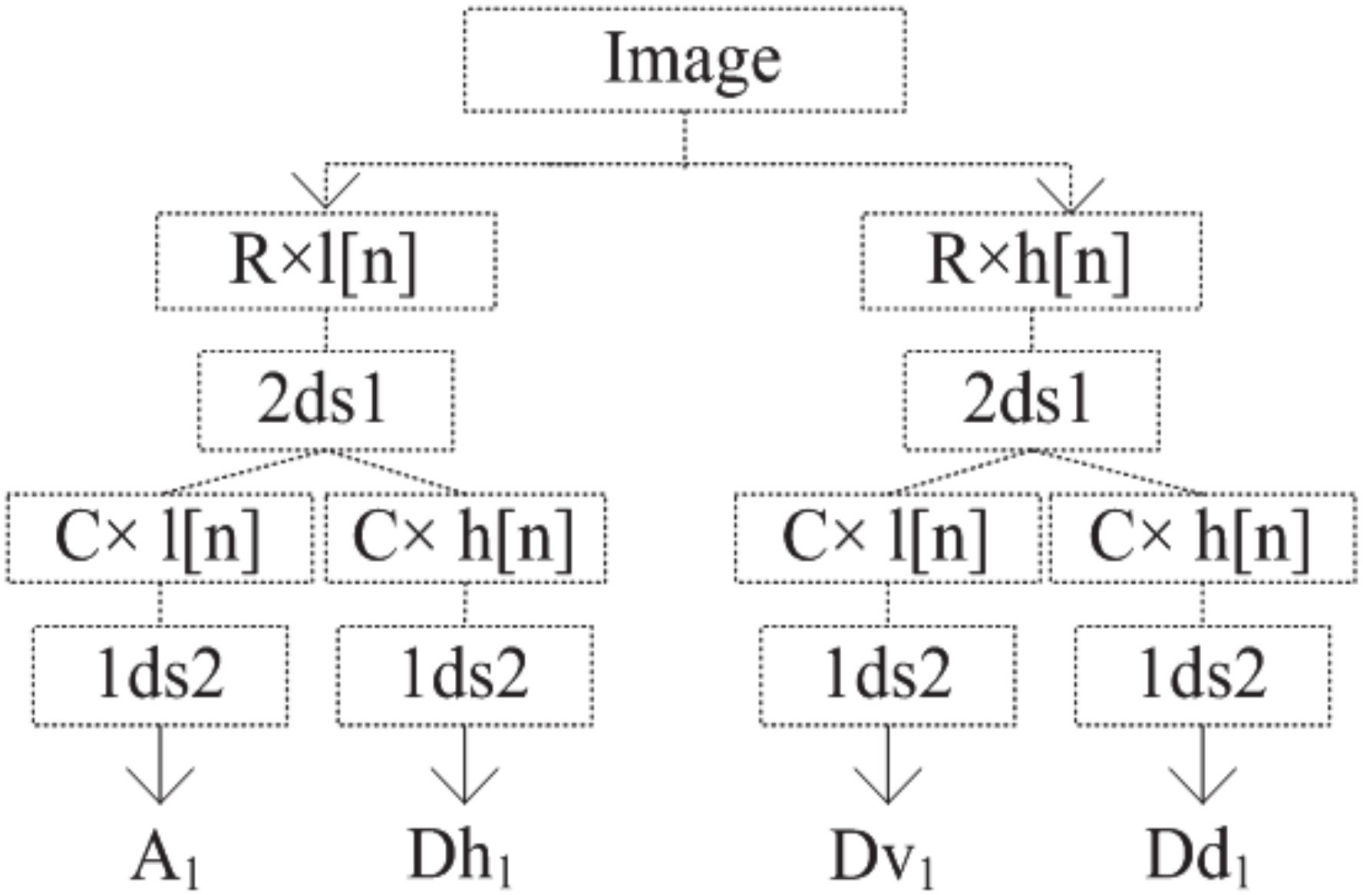
Image DW transform decomposition. R corresponds to rows, C corresponds to columns, l and h are the index of low and high pass filter respectively, 2ds1 and 1ds2 are the down-sample columns and rows respectively, and {×} is the convolution operator.

A one level 2D DWT decomposition was applied on each ROI, encoding the region’s texture as a low-frequency component A_1_ and three high-frequency components: D_d1_ (diagonal detail), D_v1_ (vertical detail), and D_h1_ (horizontal detail) (Fig 5). Three quantifier functions, measuring entropy (*f*_1DW_), energy (*f*_2DW_) and variance (*f*_3DW_), were applied to the average DWT coefficients matrix (i.e., the average of A_1_, Dh_1_, Dv_1_ and Dd_1_ matrices) of Daubechies (db), Coiflet (coif) and Symlet (sym) filters, respectively. For each sample, 9 DW-based texture features were obtained, corresponding to the following feature vector:

$$F_{DW}=\left\{f_{1}{}_{\text{Dw}\_\text{db}},f_{1}{}_{\text{DW}\_\text{coif}},f_{1}{}_{\text{DW}\_\text{sym}},f_{2}{}_{\text{DW}\_\text{db}},f_{2}{}_{\text{DW}\_\text{coif}},f_{2}{}_{\text{DW}\_\text{sym}},f_{3}{}_{\text{DW}\_\text{db}},f_{3}{}_{\text{DW}\_\text{coif}},f_{3}{}_{\text{DW}\_\text{sym}}\right\}$$ (8)

#### Texture based gray level co-occurrence matrix

Proposed by Haralick in [32], the gray-level co-occurrence matrix (GLCM) is an efficient texture analysis method which uses second-order statistics to characterize the properties of two or more pixel values occurring at specific locations relative to each other. Formally, GLCM matrices represent the probabilities *P*_*d*,*θ*_ (*i*, *j*) of transition from a pixel with intensity *i* to a pixel of intensity *j* separated by a translation vector defined by direction *θ* and offset *d* (also known as distance). Given a 2D image *I* of size *N* × *N*, the co-occurrence matrix *P*_*d*,*θ*_ (*i*, *j*) can be defined as

$$P_{d,\theta }\left(i,j\right)=\Sigma_{x=1}^{N}\Sigma_{y=1}^{N}\{\begin{array}{l} 1,\\ 0, \end{array}\;\begin{array}{r} if\;I\left(x,y\right)=i,\;and\;I\left(x+dx,y+dy\right)=j\\ otherwise \end{array}$$ (9)

where *dx* and *dy* specify the distance between the pixel of interest and its neighbor along the x-axis and the y-axis of an image, respectively. The GLCM matrix has a size of *Ng* × *Ng*, where *Ng* is the number of gray levels in the image.

We extracted the GLCM features as follows. A histogram equalization was first applied on the segmented ROIs encoded using 256 grey levels. GLCM matrices were then calculated based on four offsets (1, 2, 3 and 4 pixels) and four phases (0°, 45°, 90°, 135°). Using this technique, we obtained 16 GLCM matrices of size 256 × 256 for each segmented ROI. Texture features were then calculated by applying 12 quantifier functions on each GLCM matrix, and averaging the values across the 16 GLCM matrices. The 12 quantifier functions, proposed by Haralick et al. [32] and Clausi D. [33], are reported. Texture features based on the quantification of GLCM values can be expressed as the following vector:

$$F_{GLCM}=\left\{f_{1},f_{2},f_{3},f_{4},f_{5},f_{6},f_{7},f_{8},f_{9},f_{10},f_{11},f_{12}\right\}$$ (10)

#### Statistical analyses and classification criteria

A statistical analysis based on the Kruskal-Wallis test was carried out to measure the significance of texture features for discriminating between the three PT types. Features having a *p*-value of 0.01 or less were considered as statistically significant. Note that this analysis is not used for feature selection and, consequently, does not bias the following classification step.

Four classification methods were tested: linear discriminant analysis (LDA) [34], naïve Bayes (NB) [35], decision trees (DT) [36] and nearest neighbors (NN) classifier [37]. In LDA, the probability distribution functions (PDF) of classes are assumed to be multivariate Gaussian with different mean but same covariance matrix, and Bayes classification is used to select the class with maximum probability for each test sample. Naïve Bayes considers features as independent from one another, given their class, and computes the PDF parameters of these features for each class. A univariate Gaussian PDF is assumed for all features. DT classification splits the set of training samples recursively, by applying a threshold on selected features, until all leaf nodes are sufficiently pure (i.e., they contain samples of the same class) or a maximum number of levels is attained. In this work, the Gini index was used as measure of purity [38]. NN finds the *K* training samples nearest to a given test sample, based on the Euclidean distance, and assigns the test sample to the most frequent class of its nearest neighbors. Based on prior testing, a value of *K* = 10 was used for the number of nearest neighbors.

Classifier performance is evaluated using three metrics: accuracy, sensitivity, and specificity [39]. Accuracy measures the proportion of test samples correctly classified by the method. Sensitivity computes, for each class, the ratio between the number of true positives (i.e., positives samples classified as positive by the method) and total number of positive samples. The values obtained for each class are then averaged proportionally to the number of samples in the corresponding class. Likewise, specificity is the proportion of negative samples that are classified as negative by the method. In addition, the area under the receiver operating characteristic curve (AUC) is used to evaluate the classifiers’ performance for different decision thresholds. AUC values are obtained by plotting the curve of true positive rate (i.e., sensitivity) versus false positive rate (i.e., 1-specificity), for various decision thresholds, and measuring the total area under the curve. A higher AUC value indicates a better classifier.

A 10-fold cross-validation approach was employed to obtain unbiased estimates of classifier performance. In this approach, the data is first partitioned into 10 equal-sized sample sets. Each set is then held-out in turn for validation, while the remaining samples are used for training [40]. The average performance, computed over these 10 folds, is reported.

## Results

### Segmentation

Table 1 shows the segmentation accuracy in terms of DSC, FPR and FNR obtained by the active contour segmentation method, for images of the three PT types. We observe DSC values in the range of 86.31%–88.21%, with the best performance achieved for Ca regions. Moreover, ranges of 5.03%–7.61% and 16.11%–20.26% were obtained for FPR and FNR, respectively, the lowest values corresponding to IN regions (FPR = 5.03%) and Ca regions (FNR = 16.11%). These results confirm the ability of active contour segmentation to accurately extract the ROIs, in particular, regions corresponding to Ca.

**Table 1:** Average performance metrics (%) for the three pathological tissue types.

Metrics	BH	IN	Ca
**DSC**	86.44	86.31	88.21
**FPR**	07.61	05.03	06.32
**FNR**	18.08	20.26	16.11
**Image number**	160	144	176

### Texture analysis

The mean and standard deviation of LoG-based texture features, obtained at different filter scales, are shown in Table 2. A Kruskall-Wallis test was used for each feature to determine if its distribution of values differs across PT types. In this test, the null hypothesis is that the mean rank of values is the same for each type. The *p*-values obtained for each feature are given in the last column of Table 2. Except for *A*_*c*_, the mean rank of LoG features is significantly higher in Ca than IN and BH (*p*-value < 0.0001). Likewise, the mean rank of IN is higher than BH, except for features *A*_*c*_ and *Ent*_*f*_. This supports the idea that PT types have different textural properties and that features based on LoG can be used to discriminate between these types of tissue abnormality.

**Table 2:** Mean (± standard deviation) of LoG texture features at different PT types.

LoG based feature	BH (n = 160)	IN (n = 144)	Ca (n = 176)	*p*-value
***A***_***f***_	0.511 ± 0.014	0.513 ± 0.014	0.533 ± 0.016	< 0.0001
***Ent***_***f***_	0.031 ± 0.006	0.030 ± 0.003	0.041 ± 0.004	< 0.0001
***SD***_***f***_	4.251 ± 0.505	4.406 ± 0.447	5.178 ± 0.269	< 0.0001
***A***_***m***_	0.538 ± 0.021	0.551 ± 0.031	0.572 ± 0.026	< 0.0001
***Ent***_***m***_	0.047 ± 0.009	0.049 ± 0.010	0.070 ± 0.008	< 0.0001
***SD***_***m***_	5.032 ± 0.577	5.359 ± 0.481	6.091 ± 0.243	< 0.0001
***A***_***c***_	0.517 ± 0.047	0.529 ± 0.060	0.503 ± 0.027	< 0.0001
***Ent***_***c***_	0.064 ± 0.011	0.069 ± 0.010	0.088 ± 0.010	< 0.0001
***SD***_***c***_	5.502 ± 0.538	6.009 ± 0.304	6.459 ± 0.203	< 0.0001

A similar analysis was conducted for GLCM features (Table 3) and DW features (Table 4). For GLCM, the mean rank of features *f*_1_, *f*_2_, *f*_4_, *f*_8_, *f*_11_ and *f*_12_ is significantly higher in Ca than IN and BH types. In contrast, the mean rank of feature *f*_*9*_ is higher in IN compared to BH and Ca. Additionally, the mean rank of features *f*_5_, *f*_6_, *f*_7_ and *f*_10_ was found to be significantly higher in BH than IN (*p*-value < 0.0001). For features extracted using the DW transform, we found that features *f*_1DW_db_, and f_1DW_sym_ had a higher mean rank for IN than BH and Ca types, and that the mean rank of features *f*_1DW_coif_, f_2DW_db_, *f*_2DW_coif_, *f*_2DW_sym_, *f*_3DW_db_, *f*_3DW_coif_ and *f*_3DW_sym_ was higher in BH than IN and Ca types (*p*-value < 0.001).

**Table 3:** Mean (± standard deviation) of texture features extracted from GLCM of the different PT types.

Feature based GLCM	BH (n = 160)	IN (n = 144)	Ca (n = 176)	*p*-value
**Energy (*f***_***1***_**)**	0.051 ± 0.092	0.003 ± 0.005	0.006 ± 0.017	< 0.001
**Entropy (*f***_***2***_**)**	6.294 ± 1.572	7.137 ± 0.807	8.072 ± 0.647	< 0.001
**Correlation (*f***_***3***_**)**	0.984 ± 0.026	0.984 ± 0.006	0.966 ± 0.008	< 0.001
**Contrast (*f***_***4***_**)**	46.4 ± 24.7	54.2 ± 37.2	136.152 ± 42.164	< 0.001
**Inverse difference (*f***_***5***_**)**	0.986 ± 0.005	0.983 ± 0.006	0.970 ± 0.005	< 0.001
**Sum-variance (*f***_***6***_**)**	1.5×10^5^ ± 3.6×10^4^	1.1× 10^5^ ± 4.3 ×10^4^	1×10^5^ ± 4.2×10^4^	< 0.001
**Sum-mean (*f***_***7***_**)**	379.195 ± 54.045	331.279 ± 71.136	314.309 ± 68.274	< 0.001
**Difference entropy (*f***_***8***_**)**	2.187 ± 0.568	2.479 ± 0.380	3.082 ± 0.187	< 0.001
**Cluster shade (*f***_***9***_**)**	-3.5×10^5^ ± 4.3×10^5^	1.6×10^5^ ± 7×10^5^	7.5 10^4^ ± 5.7 10^5^	< 0.001
**Cluster tendency (*f***_***10***_**)**	2.7×10^8^ ± 1.6×10^5^	1.9×10^8^ ± 1.6×10^8^	2.1×10^8^ ± 1×10^8^	< 0.001
**Maximum probability (*f***_***11***_**)**	0.123 ± 0.164	0.020 ± 0.032	0.036 ± 0.068	< 0.001
**Difference variance (*f***_***12***_**)**	46.406 ± 24.764	54.209 ± 37.287	136.152 ± 42.164	< 0.001

**Table 4:** Mean (± standard deviation) of DW based feature at the different PT types.

DW based feature	BH (n = 160)	IN (n = 144)	Ca (n = 176)	*p*-value
***$f_{1}{}_{{}_{DW\_db}}$***	0.96 ± 0.09	1.01 ± 0.03	0.95 ± 0.03	< 0.001
***$f_{1}{}_{{}_{DW\_coif}}$***	2.65 ± 0.35	2.06 ± 0.28	2.03 ± 0.2	< 0.001
***$f_{1}{}_{{}_{DW\_sym}}$***	2.48 ± 0.309	2.5 ± 0.24	1.9 ± 0.2	< 0.001
***$f_{2}{}_{{}_{DW\_db}}$***	1.67×10^9^ ± 2.32×10^9^	1.3×10^9^ ± 1.87×10^9^	1.43×10^9^ ± 1.8×10^9^	< 0.001
***$f_{2}{}_{{}_{DW\_coif}}$***	1.67×10^9^ ± 2.32×10^9^	1.3×10^9^ ± 1.87×10^9^	1.43×10^9^ ± 10^9^	< 0.001
***$f_{2}{}_{{}_{DW\_sym}}$***	1.67×10^9^ ± 2.31×10^9^	1.3×10^9^ ± 1.87×10^9^	1.43×10^9^ ± 10^9^	< 0.001
***$f_{3}{}_{{}_{DW\_db}}$***	1578.4 ± 2486.5	1012.5 ± 1479.2	11549.9 ± 1603	< 0.001
***$f_{3}{}_{{}_{DW\_coif}}$***	1578.4 ± 2501.6	1012.5 ± 1502.2	11549.9 ± 1649	< 0.001
***$f_{3}{}_{{}_{DW\_sym}}$***	1578.4 ± 2495.417	1012.5 ± 1498.4	11549.9 ± 1641	< 0.001

Overall, the analysis shows the potential of LoG, GLCM and DW texture features for differentiating between PT types. Furthermore, since all texture features were found to be statistically significant (*p*-value < 0.01), all of them were used for classification (i.e., no feature selection step was performed prior to classification).

### Classification

The three sets of texture features (i.e., *F*_LOG_, *F*_DW_ and *F*_GLCM_) were evaluated in a classification setting, using them as input to LDA, NB, DT and NN classifiers. The performance, in terms of accuracy, sensitivity and specificity, obtained by these classifiers for each feature set is reported in Table 5. The best classification accuracies obtained for LoG, DW and GLCM are 81.17% (DT classifier), 90.00% (LDA classifier) and 94.37% (LDA classifier), respectively.

**Table 5:** Summary of image classification: 160 BH, 144 IN and 176 Ca.

Texture	Feature set	Classifier	Accuracy (%)	Sensitivity (%)	Specificity (%)
**LoG**	*F*_LoG, 9 features_	LDA	79.74	70.63	96.05
		DT	81.17	78.75	89.80
		NB	68.10	53.75	89.80
		KNN	75.43	79.37	85.86
**DW**	*F*_DW, 9 features_	LDA	90.00	80.63	96.88
		DT	86.46	87.50	95.00
		NB	61.04	41.88	96.88
		KNN	50.83	61.25	75.31
**GLCM**	*F*_GLCM, 12 features_	LDA	94.37	95.63	100
		DT	90.00	93.13	94.37
		NB	72.71	48.75	95.31
		KNN	51.67	52.50	73.75
**Concatenation**	*F*_Full feature set, 30 features_	LDA	98.92	98.12	99.67
		DT	89.87	91.87	95.39
		NB	71.34	50.00	95.07
		KNN	53.02	59.38	75.99

Concatenation: combined all the features derived from LoG, DW and GLCM features.

**Fig 6:**
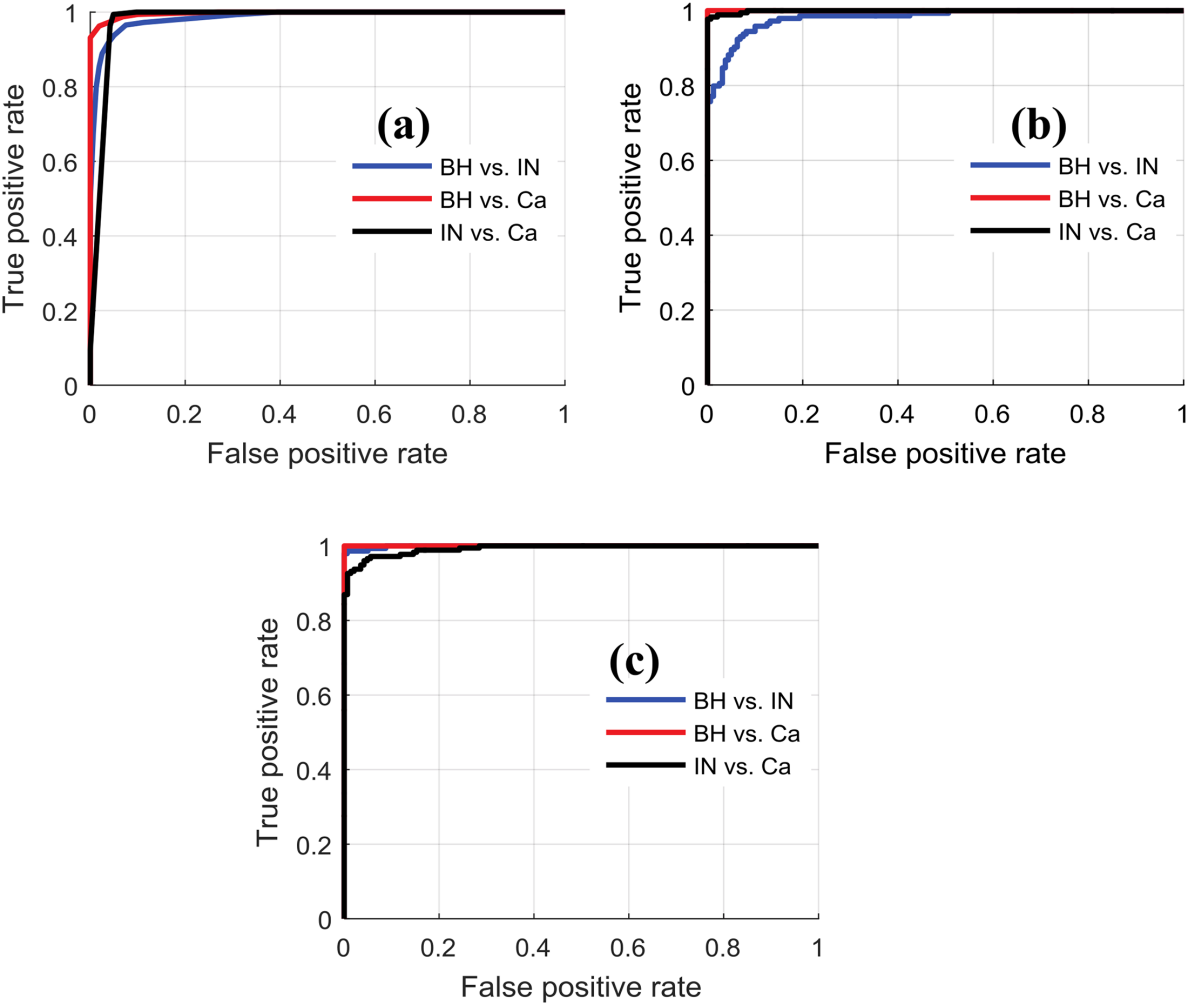
ROC analysis for the assessment of continuum PT. The blue, black, and red line is for BH vs. IN, BH vs. Ca, and IN vs. Ca respectively. (a) Texture based on LoG filter. (b) Texture based on DWT filter. (c) Texture based on GLCM.

The ability of the classifiers to discriminate between pairs of PT types, for various decision thresholds, was evaluated using the AUC metric. Results, shown in Table 6 and Fig 6, indicate that all three sets of texture features are useful to discriminate between all pairs of PT types, with AUC values ranging from 98% to 100%. Moreover, Table 7 gives the confusion matrix obtained for the three types of texture features. We see that, for all texture types, the highest accuracy is achieved for Ca (167/176 correctly classified Ca samples using GLCM), and the most frequent classification error occurs between BH and IN types.

**Table 6:** Summary of AUC metrics.

Texture technique	Nb of features	Classifier	BH vs. IN	BH vs. Ca	IN vs Ca
**LoG**	9	DT	98.57	99.69	98.00
**DWT**	9	LDA	98.07	100	99.90
**GLCM**	12	LDA	99.90	100	99.26
**Concatenation**	30	LDA	100	100	100

**Table 7:** Summary of confusion matrix.

Texture type	LoG			DW			GLCM			Concatenation		
Classifier	DT			LDA			LDA			LDA		
PT type	BH	IN	Ca	BH	IN	Ca	BH	IN	Ca	BH	IN	Ca
**BH (n = 160)**	126	25	9	129	30	1	153	7	0	157	1	2
**IN (n = 144)**	19	113	12	10	132	2	0	133	11	1	143	0
**Ca (n = 176)**	12	15	149	0	5	171	0	9	167	0	1	175

To further improve the classification performance, we concatenated the features derived from all three types of texture, giving a vector of 30 texture features. Using this approach, we obtained an accuracy of 98.92%, a sensitivity of 98.12%, a specificity of 99.67%, and an AUC of 100% using the LDA classifier (Tables 5 and 6). The performance improvement obtained with multi-texture features can also be observed in the confusion matrix of Table 7, with 157/160 of BH, 143/144 of IN, and 175/176 of Ca samples correctly classified.

### Randomization test

Randomized permutation tests were used to further quantify the significance of the link between image textures and PT types. Multiple trials were conducted to compute the classification accuracy from randomly permuted PT type labels. This approach allows the quantification of the null distribution of texture feature classification accuracy, i.e. given the null hypothesis that features contain no information regarding PT types, see permutation testing [41]. The analysis was performed as before, except that type labels were randomly permuted prior to evaluation, thereby generating an empirical null distribution over classification results from multiple trials (1000 times). As expected, the null distribution is peaked around classification values equivalent to random guessing. e.g. accuracy = 33.25% (median = 32.86%) for texture features based on LoG filter, 33.81% (median = 33.75%) for features based on DW, 33.83% (median = 33.95%) for the features derived from GLCM, and 33.91% (median = 33.93%) for full feature set (combined features), (Table 8). These distributions can be used to calculate empirical *p*-values for classification results obtained in experiments in Section 3, e.g. Table 5, which are in the significant range.

**Table 8:** Summary of randomization test.

Texture technique	Nb of features	Average ± stdev	Median
**LoG**	9	33.25 ± 2.94	32.86
**DW**	9	33.81 ± 3.08	33.75
**GLCM**	12	33.83 ± 3.19	33.95
**Full feature set**	30	33.91 ± 3.11	33.93

## Discussion

A multispectral image processing pipeline was presented, in which regions of interest (ROIs) representing abnormal tissues are automatically segmented via an efficient multi-resolution active contour method. This method was shown to be accurate, with respect to an expert labeled ground truth, obtaining Dice similarity values between 86.31% and 88.21%.

In a comparative study, we evaluated the usefulness of three types of textures for classifying pathological tissues related to CRC. Individually, all textures lead to classification accuracies above 80%, although GLCM based textures provided the best performance with an accuracy of 94.37%, sensitivity of 95.63% and specificity of 100% (Table 5). Comparing the performance across PT types, we observed that Ca samples have the lowest error rate, and that most errors occurred between BH and IN types (Table 7). We also observed that combining all three texture types (for a total of 30 features) provides the best performance, with an accuracy of 98.92%, sensitivity of 98.12%, specificity of 99.67% and AUC of 100% (Tables 5, 6 and 7). This indicates that different texture features contain complementary information, which can be combined in a synergistic fashion to improve the analysis.

**Fig 7:**
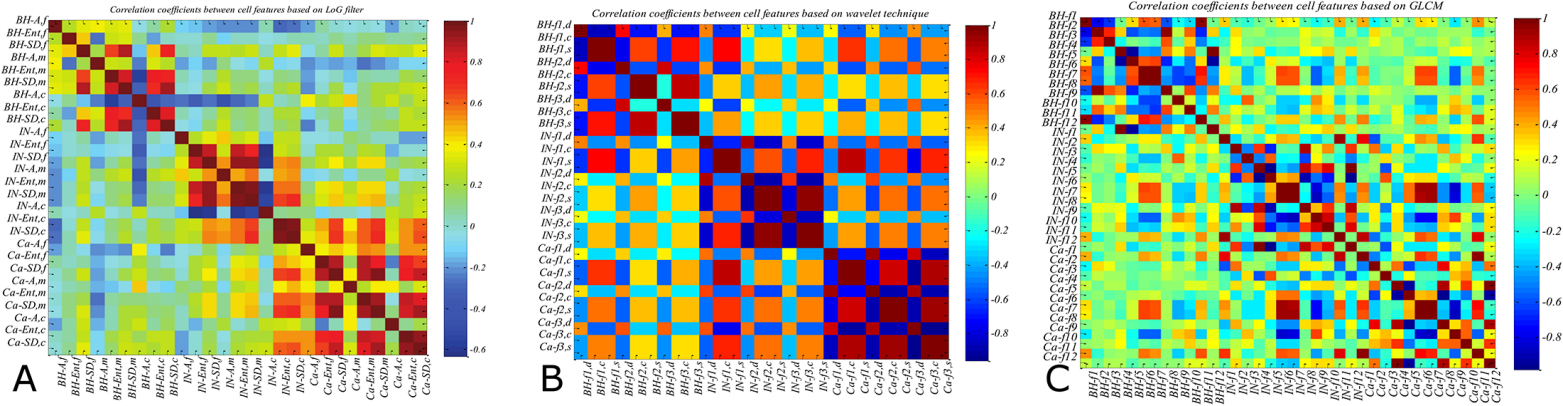
Heatmap of correlation coefficients between PT features: BH, IN, and Ca is the begin hyperplasia, intraepithelial neoplasia and carcinoma respectively. (a) Texture feature based on LoG filter, *A*, *Ent* and *SD* is the Average, Entropy and Standard Deviation respectively; *f*, *m* and *c* is the index of fine, medium and coarse texture respectively. (b) Texture feature based on discrete wavelet where *f*_*1*_, *f*_*2*_, *and f*_*3*_ is the Entropy, Energy and Variance respectively; *d*, *c* and *s* is the index of Daubechies, Coiflet and Symlet wavelet respectively. (c) Texture feature extraction from GLCM where *f*_*1*_, *f*_*2*_, *f*_*3*_, *f*_*4*_, *f*_*5*_, *f*_*6*_, *f*_*7*_, *f*_*8*_, *f*_*9*_, *f*_*10*_, *f*_*11*_ *and f*_*12*_ is the index of Energy, Entropy, Correlation, Contrast, Inverse difference, Sum-variance, Sum-mean, Difference entropy, Cluster shade, Cluster tendency, Maximum probability, and Difference variance respectively.

Fig 7 shows the correlation between the feature values found in the three PT types, for each type of texture. For LoG based texture features, the highest correlation values are observed between the medium and coarse textures of IN and Ca types (Fig 7A). A similar correlation pattern is found between the wavelet features within the IN and Ca types (Fig 7B). In contrast, less correlation is observed between different GLCM features (Fig 7C), which could explain their relatively high classification accuracy. Across PT types, low correlation values are observed between features, especially for LoG and GLCM textures. Once more, this supports the driving hypothesis that texture features can be used to characterize and identify pathological tissues in multispectral CRC images.

Finally, we note that several studies in the literature have outlined the advantages of using texture features to identify abnormal colon samples [6, 9, 10, 42, 43, 44]. However, few of them have focused on distinguishing cancer grades using the progression of pathological tissues. One such study uses multiscale local binary patterns (LBP) and support vector machines (SVM) to analyze colorectal tumor biopsies, reporting a classification accuracy of 91.28% [22]. Deep learning methods have also been used for both classification and feature learning in colon histopathology images, achieving an accuracy of 97.30% for classifying between cancer and non-cancer images [45]. While such studies did not use multispectral images nor considered the continuum of CRC (i.e., various PT types), as in our work, deep learning methods like convolution neural networks (CNN) show a great promise in improving the analysis of CRC. In particular, these methods would allow the analysis of texture at various image scales.

## Conclusion

Multispectral texture analysis is a promising noninvasive approach to quantify the spatial heterogeneity of pathological tissues (PT) associated with CRC. Our results suggest that such analysis can help detect PT in the progression of CRC from benign cell proliferation to malignant lesions. Future work will further investigate the relationship between image texture features and CRC. In particular, using a larger variety of tissue samples and cellular abnormalities would help evaluate the generalizability of the proposed method, possibly in the context of other types of cancer. Moreover, since texture features can be acquired from arbitrary imaging modalities, the usefulness of our texture-based analysis approach could also be tested on modalities other than optical microscopy, such as MRI or CT.

## Supporting Information

S1 Zip Multispectral images. BH: Begin Hyperplasia; IN: Intraepithelial Neoplasia; Ca: Carcinoma.
(ZIP)
